# Selectively-Packaged Proteins in Breast Cancer Extracellular Vesicles Involved in Metastasis

**DOI:** 10.3390/ijms21144990

**Published:** 2020-07-15

**Authors:** Penelope V. Dalla, Jerran Santos, Bruce K. Milthorpe, Matthew P. Padula

**Affiliations:** 1 Respiratory Cellular and Molecular Biology, Woolcock Institute of Medical Research, The University of Sydney, Sydney, NSW 2037, Australia; 2 Advanced Tissue Engineering and Stem Cell Biology Group, School of Life Sciences, Faculty of Science, University of Technology Sydney, Sydney, NSW 2007, Australia; Jerran.Santos@uts.edu.au (J.S.); Bruce.Milthorpe@uts.edu.au (B.K.M.); 3 Proteomics Core Facility, School of Life Sciences, Faculty of Science, University of Technology Sydney, Sydney, NSW 2007, Australia; Matthew.Padula@uts.edu.au

**Keywords:** extracellular vesicles, metastasis, breast cancer, cytokines, proteomics

## Abstract

Cancer-derived extracellular vesicles are known to play a role in the progression of the disease. In this rapidly-growing field, there are many reports of phenotypic changes in cells following exposure to cancer-derived extracellular vesicles. This study examines the protein contents of vesicles derived from three well-known breast cancer cell lines, MCF-7, MDA-MB-231 and T47D, using peptide-centric LC-MS/MS and cytokine multiplex immunoassay analysis to understand the molecular basis of these changes. Through these techniques a large number of proteins within these vesicles were identified. A large proportion of these proteins are known to be important in cancer formation and progression and associated with cancer signaling, angiogenesis, metastasis and invasion and immune regulation. This highlights the importance of extracellular vesicles (EVs) in cancer communications and shows some of the mechanisms the vesicles use to assist in cancer progression.

## 1. Introduction

Cell-to-cell communication is essential for normal function of multi-cellular organisms, as cells are required to communicate with other cells both adjacent and farther afield. Cellular communication can occur via proteins, cytokines, hormones, nucleic acids, cell–cell junction complexes, ion channels and neurotransmitters [1,2]. Several of these molecules have also been implicated in the communication and metastasising of cancer cells. Extracellular vesicles (EVs) have been shown to have a role in cellular cross-talk and to affect the cellular microenvironment in both normal and disease states [3,4,5,6]. The ability of cancerous cells to exchange material between cells enables the dissemination of mechanisms for the tumours’ survival and proliferation [1,7,8]. Thus, a thorough understanding of cell-to-cell communications through the action of EVs is essential to identify and inhibit these mechanisms of oncogenic–cargo transfer and therefore enable the development of more effective cancer treatments.

EVs have been associated with the spread and progression of breast cancer [9,10]. EVs are membrane-bound vesicles shed from cells, that contain cargo specific to their cell of origin, including nucleic acids, proteins, metabolites and cytokines [11,12,13]. It is this capacity to carry contents from their cell of origin that allows EVs to be active in the initiation and progression of cancer in a dynamic signalling process [14]. These EVs are readily available for analysis in cell secretions from cell culture media in vitro or in bodily fluids in vivo [15] and tumour-derived EVs have been detected clinically within serum and saliva from breast cancer patients [16,17,18]. The analysis of the proteins in EVs can be used to understand the proteins expressed in cancer, and how the cargo they disseminate within the body contributes to metastasis. EVs have been demonstrated to be biologically active, and those originating from tumour cells have been shown to have a systemic effect, assisting tumorigenesis and progression [19,20]. However, the specific molecules involved in these mechanisms are not well understood. The transfer of the multidrug-resistant (MDR) protein, P-glycoprotein, via EVs, and the subsequent acquisition of an MDR phenotype in the recipient cells has been shown in breast cancer and leukemia models [21,22]. These cancer-associated proteins have been detected within EVs derived from breast cancer cell lines, yet the systemic role of these is not yet well understood [23,24,25].

The process of metastasis, like much in cancer, is not straightforward and there are multiple different pathways for metastasis. This process entails changes in a cancer cell that enable it to break away from the tumour, to migrate through different tissues, intravasate and extravasate and establish a new environment in a distant site [26,27]. A diagnosis of metastatic disease takes the 5 year survival rates from 90% to only 25% in breast cancer [28], making metastatic disease a valuable prognostic indicator. Many of the proteins associated with metastasis are therefore associated with poor prognosis and are established as, or being investigated for, their potential as biomarkers and therapeutic targets. 

In order to understand the mechanisms of the effect EVs have previously demonstrated, it is first important to have a thorough understanding of their constituents. Thus, the aim of this study was to examine the proteomic and cytokine profiles of breast-cancer-derived EVs. The cells lines T47D, MCF-7 and MDA-MB-231, which are commonly-used breast cancer cell lines in EV studies, were used for this study. The small pro-inflammatory cytokine proteins in these vesicles were analysed using a sensitive multiplex immunoassay and the other proteins were analysed via label-free quantitative (LFQ) LC-MS/MS.

## 2. Results

### 2.1. Trends in EV-Associated Cytokines and Secreted Cytokines

**Figure 1:**
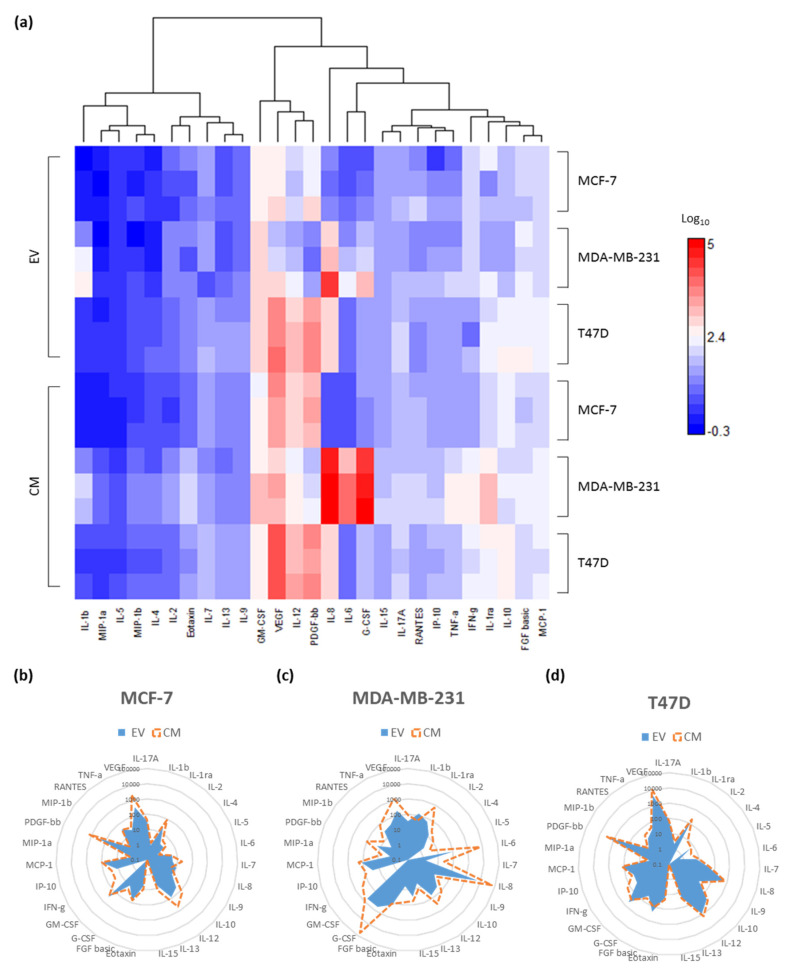
Comparisons of cytokines found in the conditioned media and associated with the extracellular vesicles (EVs) from MCF-7, MDA-MB-231 and T47D cell lines. (**a**) Heatmap depiction with hierarchical clustering using correlation clustering equation of the cytokines was carried out using DanteR software. Concentrations are in Log_10_ pg/mL. Red: high concentration, blue: low concentration and white: medium concentration of cytokines. Radar graphs comparing the mean abundance of each cytokine in each of the conditioned media, and those associated with the collected EVs for the (**b**) MCF-7 (**c**) MDA-MB-231 and (**d**) T47D cell lines (*n* = 3). The values are in picograms per millilitre and displayed on a logarithmic scale. Blue solid line: EVs and red dotted line: CM.

EVs were isolated from the breast cancer cell lines, MCF-7, T47D and MDA-MB-231, in biological triplicate. The cytokines associated with the EVs and those secreted by the same cells into the media were compared using the Bioplex multiplex immunoassay of 27-plex of inflammatory cytokines. Using DanteR software, the cytokine data were displayed in a heatmap (Figure 1a). In comparison to the other cell lines, the T47D cell-line-derived EVs had the highest concentrations of cytokines on average. The MDA-MB-231 cell line showed the highest concentrations of secreted cytokines detected in the conditioned media, followed by the T47D cell line. A positive correlation could be seen between the individual cytokines secreted into the media and those detected within the EVs of the same cell line, though those found within the EVs tended to be much lower in concentration. For instance, vascular endothelial growth factor (VEGF) has the highest detected concentration of any of the analysed cytokines in the conditioned media collected from the T47D cells (11.6 ± 1.2 ng/mL) and likewise was also found in the highest concentrations in the EVs derived from T47D cells (4.6 ± 1 ng/mL), while cytokines like IL-5 were only detected in small concentrations across both conditioned media (CM) and EV samples. The cytokines IL-8, IL-6 and granulocyte colony-stimulating factor (G-CSF) were detected in the highest concentrations in the MDA-MB-231 samples. 

To visualise the trends in EV and CM cytokines derived from the same cell line, the data were displayed on radar graphs, on a logarithmic scale (Figure 1b–d). The relative abundance of each of the cytokines for both the EV and CM, within each of the cell lines, displayed very distinct patterns. The MCF-7 and T47D cell lines showed a close correlation between the proportions of cytokines secreted in the CM and those detected within the EVs when displayed on a log_10_ axis, except IFN-γ and IL-15 in MCF-7 and IL-2 and IL-15 in T47D which were not detected within the EV samples. The MDA-MB-231s showed larger variation in cytokine abundance across EV and CM samples.

**Figure 2:**
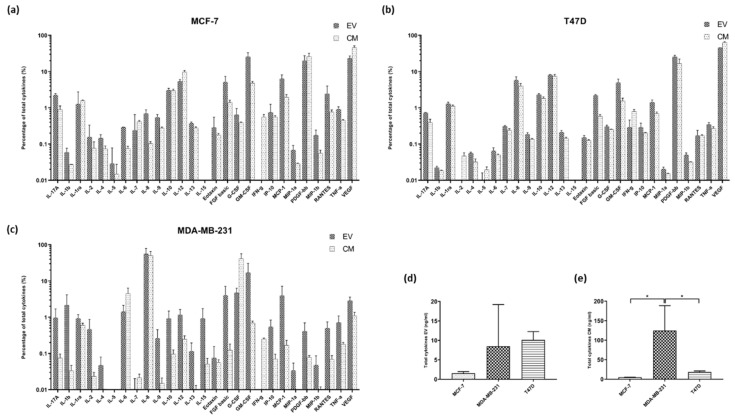
The percentage of the cytokines in each of the samples, calculated and the EVs and conditioned media (CM) for each sample directly compared to one another for the cancer lines (**a**) MCf-7, (**b**) MDA-MB-231 and (**c**) T47D on a log_10_ scale. The cytokine load of from the cells (**d**) detected in EVs, collected from a total volume of 45 mL each and (**e**) detected in the CM. A one-way ANOVA using Tukey’s multiple comparison test was conducted. Data represents means ± SD * *p* < 0.05.

In order to quantify the differences between the CM and EV, the percentage of each cytokine was then calculated across the measured cytokines for each sample (Figure 2a–c). Looking at the patterns in cytokine proportion across the panel, MCF-7 and T47D samples showed similar abundance patterns with the same cytokines being amongst the highest/lowest proportions in each sample. Conversely, the abundance in MDA-MB-231 showed very different patterns. Granulocyte-macrophage colony-stimulating factor (GM-CSF) was detected in a higher proportion in the EVs when compared to the CM across each of the cell lines by the largest amount followed by basic fibroblast growth factor (FGF basic). IL-8 was associated with MDA-MB-231, and to a lesser extent T47D EVs, at a higher proportion as with monocyte chemoattractant protein-1 (MCP-1) which is found in a higher proportion in MCF-7 and MDA-MB-231 EVs than in the corresponding CM. Platelet-derived growth factor subunits bb (PDGF-bb) was detected in a much higher proportion in the T47D EVs in comparison to the CM. Only minor proportional changes were detected in the remaining cytokines between the media and their respective EVs.

The number of cytokines from each of the EV samples was tallied, examining the total concentration of cytokines for the 27 examined cytokines (Figure 2d). In looking at the cytokines within the EVs, the T47D EVs had on average the highest detected cytokines at 10.19 ± 2.144 ng/mL, however the MDA-MB-231 had a larger diversity between the samples and the highest concentration of cytokines in a single sample (20.86 ng/mL). The MDA-MB-231 cell line media has the most identified cytokines with a mean of 125.1 ± 63.45 ng/mL and is statistically significant compared to the levels detected from the other cell lines (Figure 2e). The cells producing EVs with the next highest load of cytokines are the T47D cell secretions into the media (18.41 ± 2.688 ng/mL).

### 2.2. Label-Free Quantitative Proteomic Analysis

**Figure 3:**
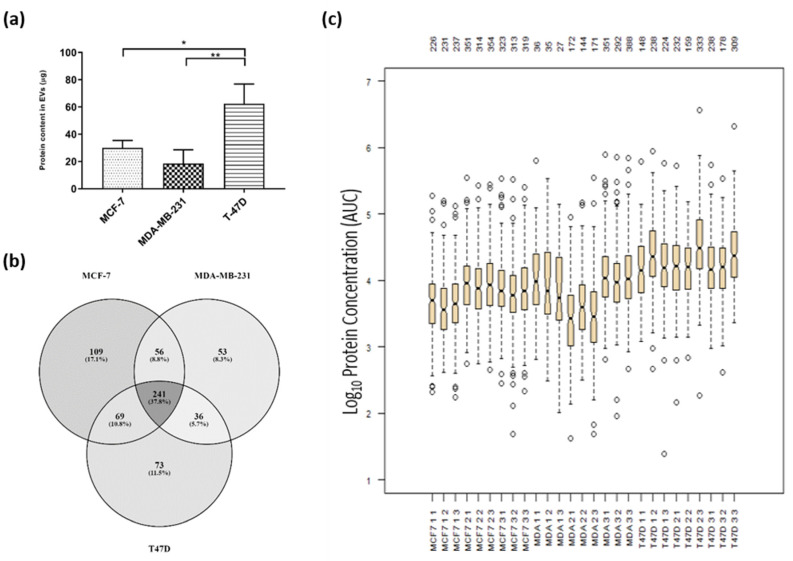
The variation between the EV proteomes across replicates and cell types. (**a**) Protein concentration of the cell-line-derived EVs using the Qubit protein assay. Data represents mean ± SD * *p* < 0.05, ** *p* < 0.01. (**b**) Venn diagram of proteins detected within EV derived from cancer cell lines. 900 proteins were detected in total with a high confidence level, having two reliable peptides or more or being identified with the same mass and retention time from the MS-1 scans. (**c**) The log_10_ of the concentration (area under the curve (AUC)) of each of the proteins are plotted in their quartiles. The number of proteins detected within each sample is shown at the top of the plot. Box and whiskers plot show the relatively small variance between the technical replicates, and the large variance between the cell of origin of the EVs. The boxplot was generated using DanteR software.

In order to profile the other proteins within the EVs, EVs from the same samples as used for the cytokine assay of the cell lines MCF-7, MDA-MB-231 and T47D were used. Following isolation, the protein content in the EV samples collected from the same volume of CM (45 mL), varied significantly (Figure 3a). There was a large range in the protein content calculated for each of the EV samples, ranging from 62 ± 14 µg in the EVs derived from the T47D cell line to 19 ± 10 µg in the EVs from the breast carcinoma cell line MDA-MB-231. 

These EVs were lysed and their proteome quantitatively analysed by LC-MS/MS using three technical replicate injections. The resulting samples were then closely examined using the Peaks Studio 8.5 software. Quantitative changes across the samples were measured using label-free quantification (LFQ) in PEAKS Studio 8.5 Q quantification software. The area under the curve (AUC) of the MS1 spectrum was used to compare the abundance of the same peptide across all samples, and the relative concentration was calculated for each sample where the protein/peptide was present. Samples without MS2 fragmentation spectra in a particular sample were then assigned an identity by having an identical peptide mass to that found in an MS1 scan at the same retention time in a different sample where the peptide had been identified from an MS2 scan and then quantified by the AUC, otherwise referred to as match-between-runs. Samples without either MS1 or MS2 spectra were not included in the relative quantitation as the peptide was not identified. Peptides identified confidently in at least one sample could therefore be used for the quantification in all samples. PEAKS then calculated each protein’s abundance as a ratio in comparison to a reference sample. This data is available in Table S2.

The proteins identified in the vesicles derived from the three cell lines were compared using a Venn diagram (Figure 3b). A total of 637 proteins were identified across the EVs of the three breast cancer cell lines. As many as 475 proteins were identified in the MCF-7 EVs, 419 in the T47D EVs and as few as 386 in the MDA-MB-231 EVs. Of the proteins identified, 38% were found in all three sample-sets. These common proteins are of interest as they may be proteins involved in vesicle formation or trafficking, proteins related to their cell or tissue of origin, or be present due to any similarities in the cancer’s phenotypes. Gene ontology analysis confirms EV location and biological function for identified protein (Figure S2). A smaller proportion of identified proteins were unique to the one cell line with the MCF-7-derived EVs having the largest number of unique proteins at 17% and the MDA-MB-231 having only 8% of the identified proteins uniquely found in their EVs. These unshared proteins are likely to represent the differences in the parent cells that these vesicles are derived from, the differences in the cancers acquired phenotypic characteristics and the inherent cellular differences.

The proteins identified in each cell-line-derived EV were then presented in individual networks (Figure S1). Seven proteins were found to be most commonly abundant across the three cell-line-derived EVs and their biological and technical replicates, actin cytoplasmic 1; pyruvate kinase; glyceraldehyde-3-phosphate dehydrogenase; 60 kDa heat shock protein, mitochondrial; ATP synthase subunit alpha, mitochondrial; sodium/potassium-transporting ATPase subunit beta-3 and voltage-dependent anion-selective channel protein 2. These proteins directly interact with 70% of the proteins in the T47D EVs, 69% of the proteins in the MDA-MB-231 EVs and 65% of the proteins in the MCF-7 EVs. Their secondary interactions accounted for 97% for the proteins in all three of the cell-line-derived EV networks. Using the String online database (version 10.5), the expected number of interactions was calculated, by the software, for a group of unrelated proteins of the same number and presented in Table S1. There was a large increase in the number of interactions in each of the EVs—each of the cancer-cell-derived EV proteins exhibited more than a two-fold increase in interactions from the expected. The MDA-MB-231 EVs had the highest fold increase from the expected at 2.52.

To determine the population differences in the detected proteins and their concentrations between samples, the data were analysed for variance. Using DanteR software, boxplots were generated for each of the samples’ biological and technical replicates. The concentration for each protein in the samples, calculated from the area under the curve in the MS1 spectra, were displayed in boxplots on a log_10_ scale (Figure 3c). These showed the large variation in the data between the proteins detected in the EVs derived from different cell lines. Variation could be seen between the EVs isolated from different passages of cells, as the biological replicates, as well as the technical replicates, run multiple times on the LC/MS-MS for analysis. This variation showed the large heterogeneity of the EVs, either isolated from the same cell line over time or from different cells. 

### 2.3. Metastasis-Related Proteins Detected in EVs

**Figure 4:**
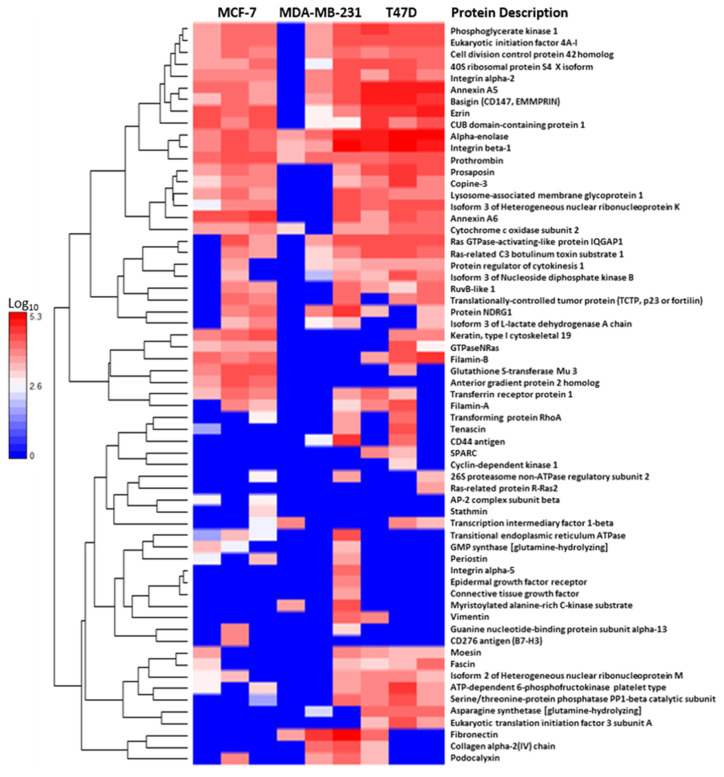
Heatmaps of the proteins associated with metastasis in cancer. Each cell represents the average of the technical replicates for one biological replicate for each of the proteins identified in the EVs derived from MCF-7, MDA-MB-231 and T47D cells. Hierarchical clustering groups the proteins with similar abundance profiles across the EV samples. The heatmaps and clusters were generated using DanteR software on a log_10_ scale.

The cancer-derived EV proteomes were examined for the presence of proteins that are known to be involved in the detrimental phenotypes associated with cancer disease and progression. There were an overwhelmingly large group of proteins associated with metastasis that were found in the data set of the vesicles from all the breast cancer vesicles. These proteins were identified using a combination of literature searching and using String online database’s Gene Ontology analysis tools. Using DanteR software these proteins were visualised in a heatmap on Log_10_ scale and a hierarchical correlation clustering algorithm in order to visualise the differential abundance of each of these samples across the biological replicates of the vesicles (Figure 4). There was a large degree of variability between the biological replicates, particularly within the MDA-MB-231 EVs which had replicates with both the fewest number of identified metastatic-related proteins and the highest. Of the metastatic proteins only alpha-enolase and integrin β-3 were identified in all biological replicates across the breast cancer vesicles. Ezrin, annexin A5, annexin A6, tenascin, CD147 and translationally-controlled tumor protein (p53) were amongst the proteins identified in each of the cell-line-derived EVs, but not all of the biological replicates. Vimentin and CD44 were identified in the EVs derived from MDA-MB-231 and T47D cells. 

## 3. Discussion

EVs derived from cancer cells have been shown to exacerbate the disease through the transfer of metabolites, nucleic acids, proteins and cytokines [29,30]. The dual approach of quantitative shotgun proteomics and cytokine immunoassay applied in this work identified a large cohort of proteins associated with the hallmarks of cancer within the EVs derived from metastatic breast cancer cells. The cytokine immunoassay allowed for the identification of a panel of analytes down to concentrations of picograms per millilitre, whereas shotgun LC-MS/MS allowed for the rapid identification of proteins but had limited ability to detect the lower abundant proteins due to the dynamic range and baseline sensitivity of the technique. These complementary techniques allowed for the identification, comparison and quantification of cytokines and proteins across the different cancers’ EVs.

### 3.1. Selective Packaging of Biologically-Significant Proteins in EVs

The contents within the analysed breast-cancer-derived EVs demonstrated selective packaging. While the process of packaging content into the vesicles is now widely acknowledged as being regulated, the mechanisms are not yet understood [31]. When examining the cytokines from the CM and EV fractions from the same cell line, despite many of the cytokines being in similar proportions in both the fractions, there were also some marked differences or even absences of certain cytokines. This suggests that some cytokines are preferentially secreted directly into the extracellular space, and that other cytokines are preferentially packaged into EVs. From the 27 examined cytokines; IL-6, IL-8, IL-12, VEGF, FGF basic, G-CSF and GM-CSF were found in higher proportions in the majority of EV samples compared to the CM from the same cell line, with FGF basic and GM-CSF found in a higher proportion in all EVs. This indicates that these are being selectively packaged into the vesicles as cargo and requires further examination. Interestingly, a study performed by Wysoczynski and colleagues demonstrated that EVs derived from cardiac mesenchymal stromal cells did not selectively package FGF basic by examining the cytokine content, indicating that the selective packaging is likely dependent on the cell type of origin of the EVs [32]. In addition to the cytokine immunoassay data, the interaction networks of the LC-MS/MS data support the theory of selected packaging as they each had 2.2-fold (or higher) more interactions within the network than a random assortment of proteins. If the vesicles contained a random assortment of proteins rather than an organised selection, the number of interacting proteins would be much lower than is seen in these vesicles. Seven proteins were found in almost all the samples in their biological and technical replicates—ACTB, PKL, GAPDH, HSPD1, ATP5A1, ATP1B3 and VDAC2. These are ubiquitous proteins, with many different roles within both healthy and cancerous cells, including cancer progression and metastasis. These seven proteins, via protein–protein interactions, connect the majority of the proteins detected within the vesicles for each cell line. It is possible that these proteins play a role in the selective packaging of the contents of the vesicles.

### 3.2. Proteins Packaged in EVs Play Roles in Metastasis

The EVs derived from cell lines in this work were shown to have very diverse proteomes, both in the number of proteins detected and protein identities. As many as 459 proteins were reliably identified across replicate samples in the EVs derived from the T47D breast cancer cell line, and as few as 374 in the EVs from the MDA-MB-231 cell line. The three cell lines examined in this study, MCF-7, MDA-MB-231 and T47D, were all derived from metastatic breast cancer, isolated from lung pleural metastases, as recorded by the ATCC cell depository, and it is unsurprising that their EVs also had metastatic related proteins. The isolated vesicles contained proteins that reflected their parental cell’s phenotype with an abundance of proteins associated with stages of metastasis. As such, the presence of selectively-packaged metastatic proteins may have a biological implication within the cells and tissues they interact with. Some studies have provided evidence that EVs have been shown to increase metastasis in in vivo and in vitro tests [33,34,35,36]. 

In the early stages of metastasis, EVs have been shown to be associated with the loss of cell adhesion, invasion and migration and with changes to the extracellular matrix [37]. This is thought to occur via the activity of vesicle-transported proteolytic enzymes that manipulate and degrade the extracellular matrix. This degradation is integral in the epithelial to mesenchymal transition (EMT) that is typically seen in the early stages of breast cancer, involving many cellular changes resulting in increased mobility and invasiveness [38]. The MDA-MB-231 and T47D EVs contain moderate amounts of tenascin, a protein known to induce EMT and the EMT marker vimentin. While it is not surprising that the MDA-MB-231 EVs contain these proteins as these cells have a mesenchymal phenotype, the T47D cells have a luminal-A breast cancer phenotype [39]. Basigin is also involved in early stages of metastasis, primarily by inducing the ECM degrading and remodeling cytokines including matrix metalloproteinases (MMPS) and VEGF. Similarly, VEGF makes changes to the ECM, stimulating the generation of blood supply via angiogenesis [40]. In addition to VEGF’s role in angiogenesis, it interacts with CD44 which in turn activates the EGFR/RAS/ERK invasion pathway [41]. Basigin and VEGF were both identified in the EVs of all the cell lines in this study and in the greatest concentrations in the T47D EVs, however CD44 was not identified in the MCF-7 EVs. VEGF expression is also stimulated by other cytokines including IL-8 and GM-CSF which were both identified in moderate–high concentrations in all EVs and clustered closely with VEGF using the correlation clustering algorithm [42,43]. IL-8 also increases invasive and metastatic potential in breast cancer and as such the overexpression of IL-8 is considered a marker for poor prognosis [44]. Conversely, Dias et al demonstrated that IL-12 activates INF-γ which in turn decreases VEGF [45]. INF-γ was identified in only low concentrations in all the EVs examined, however IL-12 was found in high concentrations in the T47D EVs, indicating a complex VEGF signaling and expression pathway.

The roles of EVs in the processes of intravasation and extravasation have been less studied, however by their nature of circulating via the vasculature they are in an ideal position to help with these stages of metastasis. In a study by Zhou et al (2014) MDA-MB-231 EVs were shown to carry miRNA that enhanced vascular permeability as required in intravasation [46]. The tumour-derived EVs examined in this study also contained a variety of proteins known to be involved in intravasation and extravasation. VEGF also manipulates the vasculature permeability [47], tenascin C is involved in changes to the cellular plasticity [48], ezrin maintains the cytoskeletal changes required for these processes [49] and podocalyxin mediates extravasation by direct interaction with ezrin [49]. These proteins were identified in all EV lines, though in higher concentrations in the MDA-MB-231 and T47D EVs. Intravasation and extravasation are key processes that lead to the final stages of colonisation and metastasis.

The colonisation of tissue by a tumour cell is a high-risk stage of metastasis with many cells going into a form of senescence or even dying due to the unfavorable environment [50]. The establishment of a non-hostile environment in the form of a pre-metastatic niche (PMN) aids in the success of this stage, and EVs have demonstrated to be highly involved in these processes [34,51,52]. The PMN formation involves the recruitment of bone-marrow-derived hematopoietic progenitor cells (and other cells). These cells are essential for the secretion of relevant proteins and recruitment of other factors to aid in the PMN establishment. Numerous tumour-derived proteins, which are known to be involved in the establishment of the PMN were identified in the breast-cancer-derived EVs examined in this study, including VEGF, TNF-α and G-CSF [52]. These cytokines are shown to be involved in the recruitment and proliferation of bone-marrow-derived hematopoietic progenitor cells, another key element of the PMN [53]. These cells preferentially localise to areas with high levels of fibronectin, which was identified in high concentrations in all three replicates in the MDA-MB-231 EVs, only one T47D EV replicate and not at all in the MCF-7 EVs. These areas of high concentrations of fibronectin are key to the PMN formation [54], which is thought to recruit other key elements to the site [55]. The high levels of fibronectin in the EVs may be a contributor to this. Fibronectin is also a ligand for CD44, which is thought to also play a key role in the evasion of apoptosis for tumour-derived cells in the early stages of colonisation [55]. The presence of these proteins and cytokines in the EVs corroborates the role EVs have in the cell-to-cell communication in PMN establishment. On the other hand, prosaposin, known to inhibit tumour metastasis [56], was identified in high concentrations in the T47D and MCF-7 EVs and only very low concentrations in the MDA-MB-231 EVs, which could contribute to the lower metastatic potential of these cell lines.

EVs are readily detected in all biological fluids [15] and therefore their applications for clinical use are vast. One of the greatest areas of focus in EV research has been the identification of biomarkers for disease diagnostics and prognosis, and for monitoring disease progression. Therapeutically, dendritic-cell-derived EVs have undergone Phase II clinical trials for their immunomodulatory effects in anti-cancer treatments [57], or as vaccines for infectious diseases in in vivo models [58]. Additionally, there have been clinical trials using EVs as drug-delivery vehicles with chemotherapeutics, for example, for cancer treatments [59]. In addition to elucidating the role of EVs in metastasis, clinically, this study has identified potential biomarkers for breast cancer metastasis to the lung that will require further validation for clinical use. Furthermore, the roles of EVs in the different stages of metastasis provide us with a unique opportunity to one day use EVs as drug-delivery vehicles to prevent the metastasis process.

The use of cell lines has allowed great discoveries in cancer biology and provided a platform for adequately exploring low abundance proteins in EVs, however their usefulness has its limitations; they do not reflect patient tumour heterogeneity and the changes cancers make over time as they progress and metastasise. Single-cell proteomics technology, such as SCoPE-MS, is evolving rapidly and reveals cellular heterogeneity [60]. Its potential applications for single-vesicle proteomics are vast and will address the problem of vesicle heterogeneity once methods of isolating single vesicles have been adequately developed.

In summary this study demonstrates that the vesicles derived from tumour cells provide powerful information about the phenotypic characteristics of cancer. There is an astounding breadth of cancer-associated proteins detected within the breast-cancer-derived EVs. The packaging of proteins into EVs allows for the transportation and trafficking of the cargo to distant sites within the body, which the cargo alone and unpackaged may not be able to disperse to on their own. This establishes cytokine communications via vesicles as another mechanism that assists in the progression of cancer and it also establishes that in the cancer-derived EVs, there is a plethora of proteins associated with the dissemination and progression of cancer. The proteomic cargo associated with the cancer-derived EVs are themselves relevant to cancer progression and thus protein trafficking and transportation is potentially one of the means by which cancer EVs affect the disease systemically.

Previous studies have demonstrated the impact of cancer-derived EVs in the processes of metastasis and this study examines the proteins and cytokines that are likely to assist in these roles. The stages of metastasis require a complex network of proteins and signaling molecules, with many redundancies. In this study, dozens of proteins known to be involved in this network have been identified in the EVs derived from the metastatic breast cancer cell lines. These proteins have been well studied for their roles in all stages of metastasis, from the initial migration to the colonising of the distal site. Some of these identified proteins have been implicated in the increased metastasis following EV transfer, however the functional transfer of many of the others still requires further investigation.

## 4. Materials and Methods 

### 4.1. Cell Culture 

The established human breast cancer cell lines MCF-7, MDA-MB-231 and T47D were kindly provided by Diana Hatoum, Eileen McGowan and Tristan Rawling. MCF-7 and T47D are classified as Luminal A type breast cancer (ER^+^, HER^+^, PR^+/−^) and MDA-MB-231 cells have a basal phenotype (ER^−^, HER^−^, PR^−^) [39]. These cells were cultured in high glucose and phenol-red-free Dulbecco’s Modified Eagle’s medium (DMEM) (Gibco, Life Technologies, Carlsbad, CA, USA), supplemented with 10% fetal bovine serum (FBS) (Gibco, Life Technologies, Carlsbad, CA, USA), 100 U/mL penicillin, 100 g/mL streptomycin and 2.5 g/mL amphotericin B (Anti-Anti) (Gibco, Life Technologies, Carlsbad, CA, USA). Cells were maintained at 37 °C with 5% CO_2_ in a humidified environment. All cells were routinely tested for *Mycoplasma* spp.

### 4.2. Conditioned Media Collection

The growth media of each cell line was collected after three days of culturing, when the cells had reached sub-confluence. The conditioned media (CM) was collected over three passages for biological replicates. CM was centrifuged at 500× *g* for 5 min to remove large cell debris and 500 µL of the supernatant was immediately frozen at −80 °C for storage until cytokine analysis. The remainder of the CM (supernatant) was used for the isolation of EVs.

### 4.3. Extracellular Vesicle Isolation

EVs were isolated from the CM by differential centrifugation as previously validated and described [22,61]. Briefly, the CM was centrifuged at 20,000× *g* for 1 h at 4 °C to pellet EVs. The pellet was resuspended in phosphate buffered saline (PBS) (Sigma-Aldrich, St. Louis, MO, USA) and centrifuged at 2000× *g* for 1 min to remove debris. The supernatant was centrifuged again 22,000× *g* for 30 min at 4 °C to pellet EVs. The EVs were resuspended in PBS and maintained at −80 °C for storage until further analysis. Concentrations of EVs were determined by protein content using Qubit protein assay (Thermo Fisher Scientific, Waltham, MA, USA) following the manufacturer’s protocol.

### 4.4. Cytokine Array

Concentrations of 27 human cytokines were simultaneously evaluated using a commercially-available multiplex bead-based sandwich immunoassay kit (BioPlex human 27-plex, catalogue number M50-0KCAF0Y, BioRad Laboratories, Hercules, CA, USA). Cytokines were collected in two fractions at the same passage from each of the cell lines in biological triplicate—secreted from the cells into the media (referred to as conditioned media or CM) and associated with the EVs. Isolated EVs were lysed, to release encapsulated cytokines, using a probe sonicator (Sonics & Materials, Inc., CT, USA) in PBS. All samples were centrifuged at 20,000× *g* for 10 min to remove any cellular debris. Assays were performed according to the manufacturer’s instructions, as previously described [62]. Images of results were generated in MS Excel, DanteR (Version 1.0.0.10, Richland, WA, USA) and GraphPad Prism (Version 8.2.1, San Diego, CA, USA). For the analysis and comparison of the EV samples, statistical analyses were performed using GraphPad Prism. The data were tested with a one-way analysis of variance (ANOVA) followed by Tukey’s multiple comparisons test. Data are represented as means ± standard deviation (SD). A *p* value of <0.05 was considered statistically significant.

### 4.5. Proteomic Preparation of EVs

EVs from the same samples analysed for their cytokine profile were also examined for the content of other proteins. The EVs were lysed in 8 M urea and 100 mM ammonium bicarbonate on ice and sonicated using a Vibra-Cell ultrasonic processor (Sonics & Materials Inc., Newtown, CT, USA). The samples were reduced and alkylated using 5 mM tributylphosphine (Sigma-Aldrich, St. Louis, MO, USA) and 20 mM acrylamide monomers (Sigma-Aldrich, St. Louis, MO, USA) for 90 min at room temperature, and the excess acrylamide was quenched with 20 mM dithiothreitol (Sigma-Aldrich, St. Louis, MO, USA). The urea was then diluted to 1 M with 100 mM ammonium bicarbonate (Sigma-Aldrich, St. Louis, MO, USA). EV lysates, ranging from 10–100 µg of protein, were digested with trypsin from porcine pancreas, proteomics grade (Sigma-Aldrich, St. Louis, MO, USA) at a 1:50 enzyme:protein ratio, and incubated at 37 °C for 16 h. Insoluble membrane components were removed by ultracentrifugation (100,000× *g* for 3 h). The digested EV proteins were de-salted using Discovery DSC-18 solid phase extraction columns (SUPELCO, Bellefonte, PA, USA). The peptide concentration was determined using the Pierce quantitative colorimetric peptide assay (Thermo Fisher Scientific, Waltham, MA, USA) and prepared for LC-MS/MS analysis.

### 4.6. LC-MS/MS 

Using an Eksigent 415 autosampler connected to a 415 nanoLC system (Eksigent, Dublin, CA, USA), 1 µg /5 µL of the samples were loaded at 300 nL/min with MS buffer A (2% acetonitrile + 0.2% formic acid) by direct injection onto a self-made column with an integrated emitter made with a laser puller [63] (75 µm ID × 150 mm) and packed with C18AQ resin (1.9 µm, 200A, Dr. Maisch, Ammerbuch, Entringen, Germany). Samples were injected in biological and technical triplicates. Peptides were eluted from the column and into the source of a 6600 TripleTOF hybrid quadrupole-time-of-flight mass spectrometer (Sciex, Redwood City, CA, USA) using the following program: 2–35% MS buffer B (80% acetonitrile + 0.2% formic acid) over 90 min, 35–95% MS buffer B over 9 min, 95% MS buffer B for 9 min and 80–2% reverse concentration gradient for 2 min. The eluting peptides were ionised at 2300 V. An intelligent data acquisition (IDA) experiment was performed, with a mass range of 350–1500 Da continuously scanned for peptides of charge state 2+–5+ with an intensity of more than 400 counts/s. Up to 50 candidate peptide ions per cycle were selected and fragmented and the product ion fragment masses measured over a mass range of 100–2000 Da. The mass of the precursor peptide was then excluded for 15 s.

### 4.7. Proteomics Data Analysis

The MS/MS raw data files were searched using PEAKS studio 8 (version 8.5, build number 20170804) (Bioinformatics Solutions, Inc., ON, Canada) against the UniProt human proteome database (www.uniprot.org). Data were imported into the program with the following parameters, enzyme: semi-trypsin, up to three missed cleavages; variable modifications: deamination (NQ), oxidation (M) and propionamide (C); maximum variable post-translational modifications per peptide: five; parent mass error tolerance: 50.0 ppm and fragment mass error tolerance: 0.1 Da. The false discovery rate (FDR) was estimated with decoy-fusion. Label-free quantitation (LFQ) values were determined using PEAKS Q using the following settings, retention time shift tolerance: 6 min, mass error tolerance: 20.0 ppm, a 1% FDR threshold was applied. Peptide filters: peptide ID ≥ 1, charge: between 1+ and 10+ and at least one confident sample. Protein filters: 2-fold change and at least one unique peptide. Following peptide identification, PEAKS LFQ algorithms used the MS1 peptide intensities to determine quantitation across all samples. The samples that did not have MS2 ionisation data were still analysed for their MS1 data, making this a robust quantification method [64]. Statistical analysis and images of results were additionally generated in Excel, DanteR (Version 1.0.0.10, Richland, WA, USA) and GraphPad Prism (Version 8.2.1, San Diego, CA, USA). Protein–-protein interaction networks were generated using Cytoscape (Version 3.7.1, Cytoscape Consortium, Seattle, WA, USA) [65]. Those accession numbers not recognised by the software were manually reassigned based on their sequence homology.

## Supplementary Materials

Supplementary materials can be found at https://www.mdpi.com/1422-0067/21/14/4990/s1.

## Abbreviations

ANOVA	Analysis of variance
ATCC	American Type Culture Collection
AUC	Area under the curve
CM	Conditioned media
EMT	Epithelial to mesenchymal transition
EV	Extracellular vesicle
FBS	Foetal bovine serum
FDR	False discovery rate
IDA	Intelligent data acquisition
LC/MS-MS	Liquid chromatography- tandem mass spectrometry
LFQ	Label-free quantitation
MDR	Multidrug resistance
MMP	Matrix metalloproteinase
MS	Mass spectrometry
PBS	Phosphate buffered saline
PMN	Pre-metastatic niche
SD	Standard deviation
