# Supplementary material for: Selectively-Packaged Proteins in Breast Cancer Extracellular Vesicles Involved in Metastasis
Source: Int J Mol Sci. 2020 Jul 15;21(14):4990. doi: 10.3390/ijms21144990 (PMC7403963; doi:10.3390/ijms21144990)
Supplement: Supplementary file 1 [file ijms-21-04990-s001.zip › suppl/Figures S1 and S2, Table S1.pdf]

International Journal of
Molecular Sciences

MDPI

Supplementary

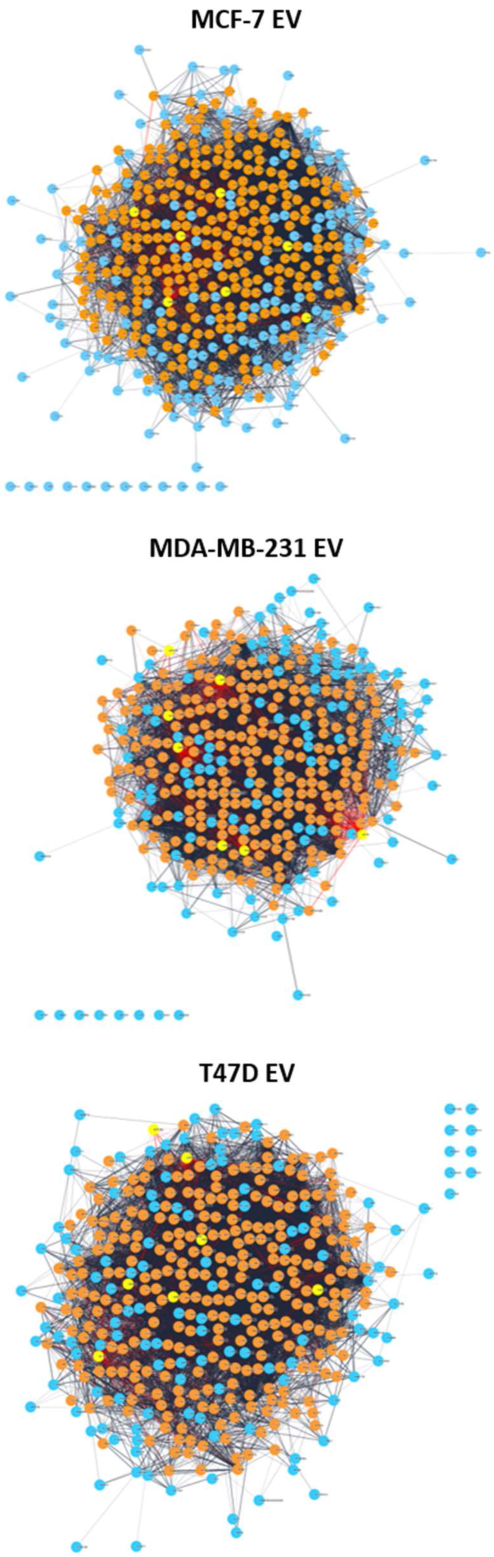

Figure S1. Interaction networks of the proteins detected within the EVs of MCF-7, MDA-MB-231 and T47D. The 7 most commonly found proteins have been highlighted in yellow, their primary interacting proteins within the network are displayed orange and the remaining protein are in blue. The networks are organised using the Organic layout algorithm using Cytoscape interaction software.

Table S1. Network interaction statistics on the proteins within EVs from each of the cell lines and within the different cell types.

Cell line derived EV	Number of proteins	Number of interactions	Expected number of interactions	Fold increase from expected
Combined EV	635	17641	7715	2.29
MCF-7	474	10449	4189	2.49
MDA-MB-231	384	8639	3424	2.52
T47D	418	8098	3587	2.26

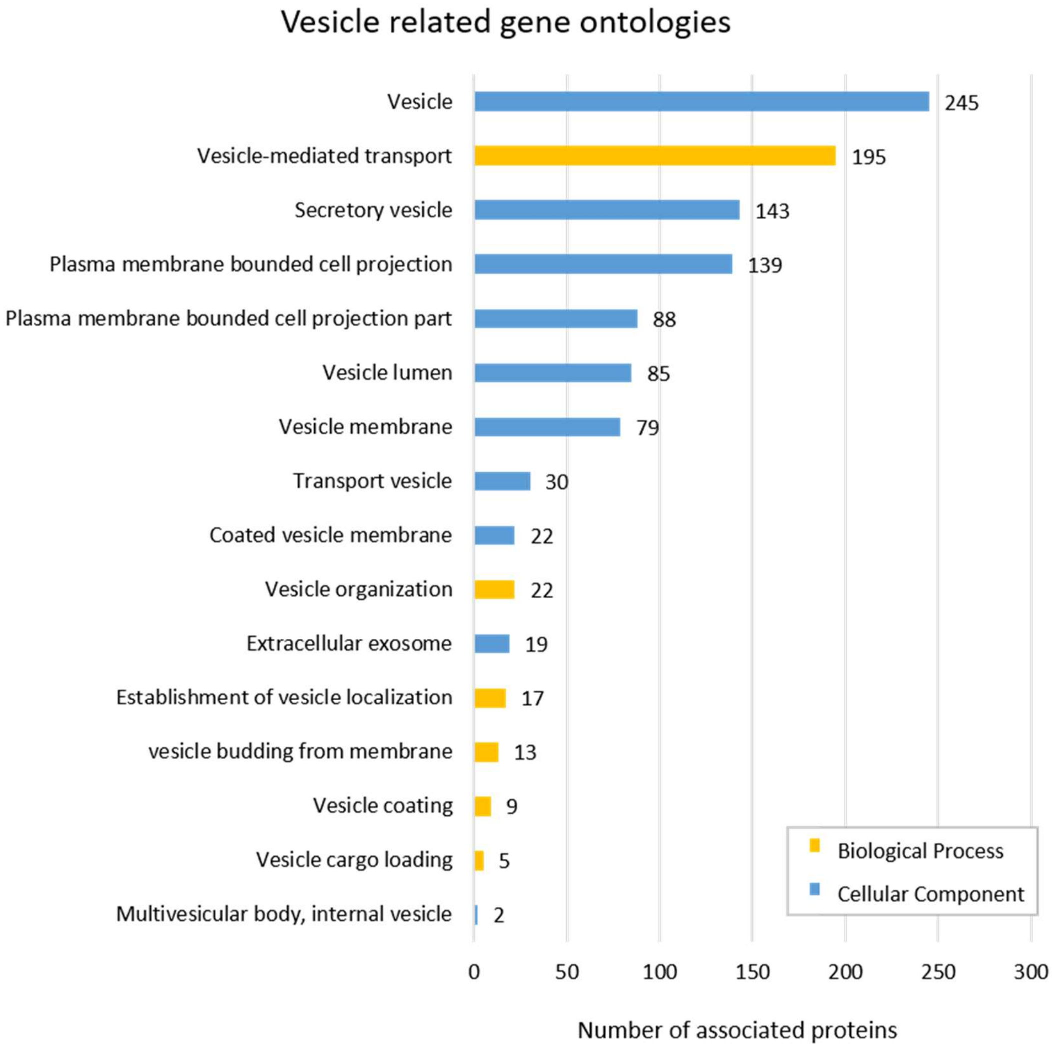

Figure S2. Vesicle related gene ontologies of the proteins detected within the EVs of MCF-7, MDA-MB-231 and T47D. The number of proteins within the data set related to each ontology is indicated to the right of each bar. Biological Process related gene ontologies are indicated in yellow and Cellular Component in blue. Gene ontologies were identified using String version 11.0 functional enrichments.
